# The use of economic evaluation in CAM: an introductory framework

**DOI:** 10.1186/1472-6882-10-66

**Published:** 2010-11-11

**Authors:** Emily Ford, Daniela Solomon, Jon Adams, Nicholas Graves

**Affiliations:** 1 School of Population Health, University of Queensland, Herston Road, Herston, Queensland, 4006, Australia; 2 School of Public Health and Institute of Health and Biomedical Innovation, Queensland University of Technology, 60 Musk Avenue, Kelvin Grove Urban Village Kelvin Grove, Queensland, 4059, Australia

## Abstract

**Background:**

For CAM to feature prominently in health care decision-making there is a need to expand the evidence-base and to further incorporate economic evaluation into research priorities.

In a world of scarce health care resources and an emphasis on efficiency and clinical efficacy, CAM, as indeed do all other treatments, requires rigorous evaluation to be considered in budget decision-making.

**Methods:**

Economic evaluation provides the tools to measure the costs and health consequences of CAM interventions and thereby inform decision making. This article offers CAM researchers an introductory framework for understanding, undertaking and disseminating economic evaluation. The types of economic evaluation available for the study of CAM are discussed, and decision modelling is introduced as a method for economic evaluation with much potential for use in CAM. Two types of decision models are introduced, decision trees and Markov models, along with a worked example of how each method is used to examine costs and health consequences. This is followed by a discussion of how this information is used by decision makers.

**Conclusions:**

Undoubtedly, economic evaluation methods form an important part of health care decision making. Without formal training it can seem a daunting task to consider economic evaluation, however, multidisciplinary teams provide an opportunity for health economists, CAM practitioners and other interested researchers, to work together to further develop the economic evaluation of CAM.

## Background

Economic evaluation is discussed often in the conventional health care literature and it is well established that clinical efficacy and cost-effectiveness evidence are necessary to assist health care decision-making within the confines of finite health budgets. Research examining the safety and efficacy of Complementary and Alternative Medicine (CAM) is rapidly growing [1], and there appears to be a potential role for CAM in a number of treatment areas [2]. In order for CAM to be extensively considered in health care decision-making there is a need to expand the evidence-base for these medicines and therapies and for the CAM research community to further incorporate economic evaluation into research priorities (alongside developing a broader health services research agenda) [3]. The aims of this article are to discuss the need for economic evaluation, outline the types of economic evaluation available for the study of CAM, and introduce decision modelling as one flexible method for economic evaluation with much potential for use in examining and assessing CAM. Our discussion offers CAM researchers an introductory framework for understanding, undertaking and disseminating economic evaluation. It is hoped that this paper will help fuel enthusiasm and develop early capacity towards producing much needed rigorous economic evaluation of CAM.

### A Growing Need for Economic Evaluation in CAM

In a world of scarce health care resources and an emphasis on efficiency and clinical efficacy, CAM (like all treatments) requires rigorous evaluation if it is to be fully considered in budget decision-making [4]. While the research base for CAM is small relative to that for conventional medicine, it is growing [1] and evidence shows some CAM therapies are safe and effective [2]. A logical next step is to examine the 'real world' impact of CAM; whether it offers value for money in comparison to, or in addition to, conventional treatments. Any informed attempt to consider CAM provision as a core component of a comprehensive and integrated health care system requires a focus upon associated change to costs and health benefits [5,6]. Economic evaluation methods provide the tools to measure the costs and health consequences of CAM interventions and thereby inform decision making.

The number of published health economic evaluations in Australia and around the world is rapidly increasing. Some researchers have identified economic evaluation and its importance in relation to CAM [7,8] and there are several systematic reviews which have examined the number and quality of economic evaluations which currently exist in the CAM literature [9,10]. One recent systematic review concluded there was a limited number of evaluations of sufficient quality to draw general conclusions. Nevertheless, this review did consider a small number of treatment areas as cost-effective compared with conventional care, including the use of acupuncture for migraines, manual therapy for neck pain, and spa therapy for Parkinson's disease. There was also evidence to suggest CAM can be considered cost effective in a complementary role in the treatment of some conditions, for example complementary guided imagery for cardiac surgery patients and complementary relaxation therapy for patients with a previous myocardial infarction. It is perhaps easier to consider that CAM approaches which are substitutes for conventional care are more likely to be cost-effective, however, there is also evidence for CAM in a complementary role, despite the addition of another treatment [6].

CAM evaluation does face potential challenges given CAM's different philosophical tenets when compared to conventional medicine [11]. Treatment is often a complex combination of several therapies tailored to the individual [1], and the therapeutic effects of CAM are often small, difficult to quantify and can occur over a long period, whether administered as a stand alone treatment or provided in addition to conventional medicine. It is important to use the evaluation framework whilst incorporating CAM-sensitive outcome measures and to focus on the development of outcome measures which capture the effects of CAM and study designs which allow these outcomes to be examined. As such, it is essential that this area be developed through collaboration between CAM practitioners and health services researchers, to ensure economic evaluation research is conducted in a manner which captures the broad benefits of CAM.

In order to address the gap in research and help others develop capacity in this significant area of CAM-related inquiry, this paper provides an overview of economic evaluation, and introduces the concept of decision modelling as a method for economic evaluation ideal for CAM.

## Methods

### Economic Evaluation for CAM

#### Different schools of thought in economic evaluation

There are two schools of thought in economic evaluation, described in brief here due to extensive description elsewhere in the CAM literature [1,6,8]. Costs are consistently measured in monetary units, benefits are measured in different ways. Cost-benefit analysis (CBA) summarises benefits in monetary values, enabling a direct comparison between costs and outcomes. Using CBA anything can be compared and health goals can be compared with non-health goals. Individual preferences are valued, often using the concept of willingness to pay (WTP). CBA can be difficult to apply as giving health outcomes monetary value can be problematic [12]. In contrast, cost effectiveness analysis (CEA) and cost-utility analysis (CUA) are useful for allocating a fixed health budget between competing treatments to maximise health improvements. CEA is used when programs or treatments have a common outcome of interest to compare. CUA is similar, except for a focus on the quality of the outcome, incorporating a sense of value or preference as captured by the use of quality adjusted life years (QALYs) [13,14]. The concept of the QALY was designed to capture changes to both quantity and quality of life from a treatment or intervention [15]. QALYs are calculated by weighting the extra time spent alive because of an intervention with a utility value, known as the QALY weight. It represents a preference based valuation of a particular health state and allows both quantity and quality to be summarised in a single measure [15,16]. The scale of QALY weights is for death to equal 0 and perfect health to equal 1. Two extra years of life in a state value at 0.7 is equal to 1.4 QALYs.

As an example of utility weights, a recently published study derived estimates for the stages of chronic heart failure, New York Heart Association (NYHA) class I, II, III, and IV had weights of 0.90, 0.83, 0.74, and 0.60 respectively. NYHA class I is an asymptomatic stage of heart failure, and so the value of that particular state is close to perfect health. On the other hand NYHA class IV is associated with persistent symptoms despite optimal medical treatment, and therefore the value of that state is further from perfect health [17]. The suitability of QALYs as a measure of outcome is a complex issue which has been discussed in both conventional and CAM literature [1,4,14,18-20]. Currently available full economic evaluations for CAM have all utilised CEA or CUA, perhaps because they are more easily applied than CBA requiring the valuation of health in monetary terms. For a comprehensive discussion of CBA versus CEA please refer to a Resources for the Future (RFF) report written by Alan Krupnick [21].

Decision analytic modelling has become increasingly popular as a method for the economic evaluation of health care. It is a flexible method which has not been previously discussed in reference to CAM. A full critique of this approach is beyond the scope of this paper, but can be found in the literature [22]. Randomised clinical trials (RCTs) can provide valuable information about treatment effectiveness and have often been used as the single source of data for economic decision making [23]. There are limitations to using only RCT data for economic evaluation. Decisions are often made between multiple competing options, which are unlikely to have been compared in a single trial. RCTs are often based on very strict criteria that reduces the ability to generalise the results. Often not all of the factors of interest are measured, intermediate measures are often used instead of final endpoints; and the timeframe for RCTs is often short, whereas lifetime costs are important when compiling economic evidence [24].

Decision analytic modelling provides an opportunity to use multiple data sources and extrapolate beyond the limits of current clinical evidence in order to include alternative interventions [25]. Other benefits include the provision of a natural structure which identifies the possible states that patients may be in and the effect of the interventions on these states; a method of transforming the evidence into estimates of costs and benefits for comparison; an assessment of the uncertainty present in the comparison of interventions; and identification of the value of future research to further inform decisions [14].

#### Decision analytic models

Development of model structure is a crucial part of the research process, the aim is to develop a model which is no more complex than it needs to be to answer the questions posed. They should be designed to represent important outcomes and estimate the relevant costs and health benefits, for each choice under consideration. Decision trees and Markov models are two common choices of models.

**Figure 1:**
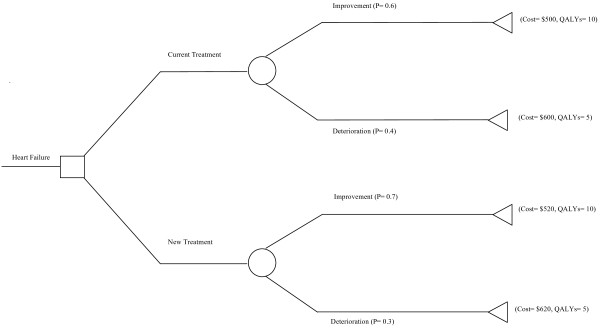
**Example of a decision tree**.

A simple decision tree is drawn in Figure 1. This represents a hypothetical case of chronic heart failure and treatment with current therapy and a new therapy comprised of the current treatment, with CAM introduced as a complement. It shows: a decision at the square 'decision node', and this is where a decision to adopt one therapy over the other is summarised; the alternate events that might occur at the circular 'chance nodes', which in this example are improvement or deterioration; and the final cost and health benefits of every possible pathway at the triangular 'terminal nodes'. The expected economic values arising from each decision are the product of the final costs and health benefits and the likelihood of the alternate events, known as probabilities. The tree is rolled back from right to left.

Probabilities for a given treatment must sum to 1, in this example for current therapy, the probability of improvement is 0.6 and the probability of deterioration is 0.4, summed together they equal 1. Probabilities are based on available information which may come from a variety of sources, including clinical trials, observational studies, case series, expert opinion, or a secondary analysis of any of the above sources [26]. The probabilities used and the quality of the data source will impact on the analysis, sensitivity analysis is performed to assess this impact [14].

There are a range of costs to consider. These include health system costs, such as drugs, hospitalisation, equipment, medical staff; costs to other sectors; patient/family costs, such as travel costs and time; and productivity costs, the effect of work time. The range of costs included in the evaluation will depend upon the viewpoint taken in the analysis. For example, travel costs incurred by the patient and patient's family members and the cost of time away from work are likely to be important from the point of view of the patient and society, however, it is may not be as important to the government [14]. There are two components of determining costs, the quantity of resources used and the market price of the resources. For example, if we consider the price of medication, conventional and CAM approaches, for heart failure and the cost of an outpatient visit to a medical practitioner, we must first establish what medications are taken, in what amounts and their price, and then how many outpatient visits a person with heart failure would have in addition to the price of the visit, or some approximation of the price. The information for this comes from a variety of sources, including published cost data, administrative databases, such as those held by the Australian Institute of Health and Welfare (AIHW), previous economic evaluations and expert opinion [26].

Calculations of expected costs and outcomes from the data drawn in Figure 1 are shown here:

Expected cost of current treatment = (0.6 × $500) + (0.4 × $600) = $540

Expected cost of new treatment = (0.7 × $520) + (0.3 × $620) = $550

Expected QALYs of current treatment = (0.6 × 10) + (0.4 × 5) = 8

Expected QALYs of new treatment = (0.7 × 10) + (0.3 × 5) = 8.5

Using the expected costs and benefits calculated, shown in Table 1, an Incremental Cost Effectiveness Ratio (ICER) can be calculated, which tells us the incremental cost per additional QALY generated by the new treatment. Information like this can be used to compare competing treatments and provide information about their value for money.

**Table 1:** Example of an ICER

Intervention	Costs	QALYs
**Current**	$ 540	8

**New**	$ 550	8.5

**Difference**	$ 10	0.5

**ICER**	$ 20	Per QALY

Calculation of the ICER = ($550-$540)/(8.5-8) = $20 per QALY.

Decision trees have limitations: time is not made explicit, and they quickly become large and unwieldy when dealing with long timeframes and multiple events, becoming visually and computationally complex.

**Figure 2:**
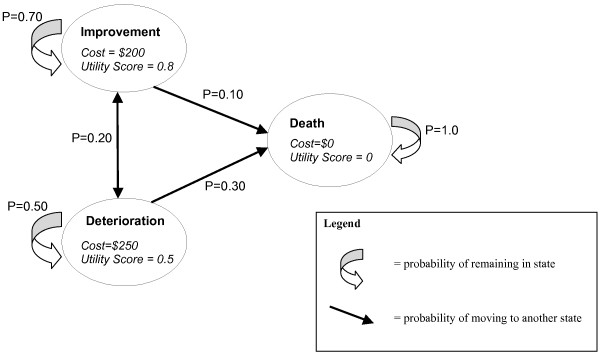
**Example of a Markov model**.

Markov models are an alternate approach to decision analytic modelling and have added flexibility and are suitable for more complex situations. There are a series of mutually exclusive 'states' drawn in Figure 2 which a patient can occupy at a given point in time instead of branches in a decision tree. Time is represented explicitly, the probability of a patient being in a particular state at a particular point in time is examined over a series of cycles. Movement between the states is determined by the calculation of transition probabilities which reflect 'real life' disease progression. Transition probabilities capture the chance of maintaining the same level of health, improvement, deterioration or death.

CAM is often complex as well as often used by those with chronic disease and those seeking long-term prevention. Markov models are ideally suited to complex chronic disease and may be ideal for the economic evaluation of CAM due to the added flexibility and ability to represent complex situations.

In Figure 2 we show the probability of a patient moving between states and remaining in states for each cycle of the model for a 'current treatment' alternative. This information is also shown as a transition matrix in Table 2. Note that the sum of each row must add up to 1, and this ensures that no patient can leave the model.

**Table 2:** Example of a Transition Matrix (current treatment)

*transition matrix*	Improvement	Deterioration	Dead
**Improvement**	*0.70*	*0.20*	*0.10*

**Deterioration**	*0.20*	*0.50*	*0.30*

**Dead**	*0.00*	*0.00*	*1.00*

Different values for the transition probabilities will be used for alternate treatments that compete with the current treatment. A new more effective treatment might for example take a lower value for the probability of moving from 'Improvement to 'Deterioration' and from 'Improvement' to 'Death'. This is shown in Table 3.

**Table 3:** Example of a Transition Matrix (new treatment)

*transition matrix*	Improvement	Deterioration	Dead
**Improvement**	*0.80*	*0.15*	*0.05*

**Deterioration**	*0.20*	*0.50*	*0.30*

**Dead**	*0.00*	*0.00*	*1.00*

Under any competing treatment alternative the movements of say 1,000 patients between health states over time (cycles) can be summarised, in this case one cycle lasts one year. Patient movements through the Markov model are shown in Table 4 for the current treatment probabilities, for the first three cycles. One thousand patients are always retained in the model.

**Table 4:** Example of Markov model cycles (current treatment)

Cycle	Improvement	Deterioration	Dead	Total
**0**	1000	0	0	1000

**1**	700	200	100	1000

**2**	530	240	230	1000

**3**	419	226	355	1000

Each row of the table represents a cycle during which time patients incur costs from treatment and accrue health outcomes shown by the utility score. Once the number of patients in each state for each cycle has been calculated, we can calculate the total costs and benefits for a treatment option. We need to know the cost and benefit associated with being in each state, which are shown in Figure 2. To calculate costs for each cycle, the number of patients in each state is multiplied by the cost of that state and then summed for all the states for that cycle. To calculate benefits for each cycle, the proportion of patients in each state is multiplied by the utility value of each state and then summed for all the states for that cycle.

The costs for current treatments in cycle 1 are (700* $200) + (200*$250) + (100*$0) = $190,000. The health benefits for cycle 1 are (700*0.8) + (200*0.5) + (100*0) = 660 QALYs. The total cost of three cycles (three years) for the current treatment is $496,300 and total QALYs is 1,652.2. When the new treatment probabilities are used in the model the costs change to $513,550 and total QALYs is 1782.7. As with the decision tree, an ICER is then calculated (see Table 5).

**Table 5:** Example of an ICER for the Markov model

Intervention	Costs	QALYs
**Current**	$496,300	1652.2

**New**	$513,550	1782.7

**Difference**	$17,250	130.5

**ICER**	$132.18	per QALY

Calculation of the ICER = ($513,550-496,300)/(1782.7-1652.2) = $132.18 per QALY.

For both modelling approaches data are required to inform probabilities of events under competing treatment options and the costs and health outcomes that arise. To identify the data that can be used to describe the models parameters a process of evidence synthesis is required.

#### evidence synthesis

Appropriate evidence for all parameters of the model needs to be identified to ensure the reliability of the model. The evidence base will consist of studies from diverse sources and the quality of evidence differs between studies. This is of particular relevance for CAM, as there are often quality issues due to difficulties with study design and the use of small samples. Evidence needs to be systematically searched and identified, and there are many documented methods for this [27]. The multiple sources of evidence include randomised controlled trials (RCTs), observational studies, cohort studies, administrative databases, expert opinion, clinical outcome registers, cost information, and routine statistics.

Evidence synthesis, the process of bringing all the information together, is a crucial step in the process of economic evaluation and decision analytic modelling. Meta-analysis is a common method for the synthesis of quantitative data, providing a numerical summary of the overall effectiveness of an intervention [28]. The quality of data included in the model will determine the model validity, especially when this data is used for parameters which have a large influence on the model results. Therefore transparency is vital when presenting the methods of economic evaluation. The reader needs to be able to see the data, the methods used for analysis and the assumptions made, in order to make an informed decision about model validity.

It is often argued that CAM is under-researched, and when evidence exists it is often of poor quality. Whilst the process of evidence synthesis and the ability to incorporate multiple data sources of varying quality do not replace rigorous research or negate the need to expand the evidence base, they do provide an opportunity to examine the current evidence base and use the data available to make a decision. The adept modeller will characterise the role of uncertainty in the decision they are informing. Methods exist, that are beyond the scope of this article, for describing uncertainty, and should be used for any applied modelling study.

### How information from models should be used by decision makers

**Figure 3:**
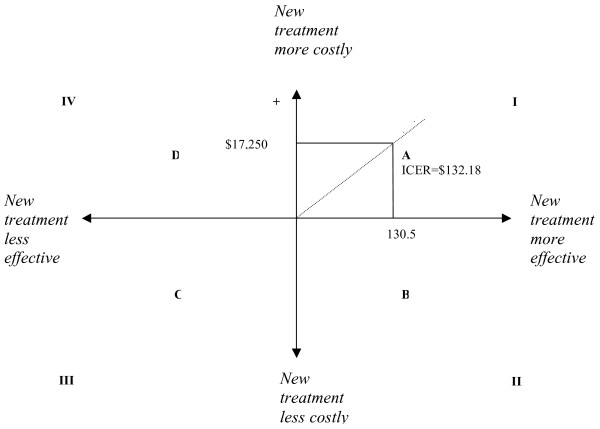
**Example of a cost effectiveness plane**.

The results of cost-effectiveness data we found from the Markov model analysis can be shown graphically using the cost-effectiveness plane (see Figure 3). In this diagram, the horizontal axis represents the difference in effect between the intervention of interest and the alternative. The vertical axis represents the difference in cost between the two interventions. The slope of the line represents the ICER (refer to Table 5). In this case the new treatment lands on a point in quadrant I (i.e., 'A') the intervention is more costly and more effective, if it lands on a point in quadrant II (ie., 'B') the intervention is less costly and more effective (it dominates the alternative), if it lands on a point in quadrant III (ie., 'C') the intervention is less costly and less effective, and if it lands on a point in quadrant IV (ie., 'D') it is more costly and less effective (it is dominated by the alternative intervention). If the intervention is located on a point in quadrant I or quadrant III a decision must be made as to whether the benefits of the more effective intervention are worth the extra costs involved.

The situation where one intervention is more effective and more costly than another occurs relatively often (ie., 'A'). If this occurs, the decision regarding whether the benefits of the more costly intervention are worth the extra costs will depend upon the threshold for the decision maker or the willingness-to-pay per unit of health gain (such as per QALY or per life year). There are many suggestions in the literature as to what constitutes an appropriate threshold value, but in reality this is an arbitrary figure and decision makers do not use such an absolute threshold [29,30]. It is necessary to have some threshold guidance for health care decisions, as the supply of new and effective technologies increases and the access to health care improves, there will be increased pressure on the health care budget which requires a consistent method for determining which interventions or therapies are worth funding [31]. The National Institute for Clinical Excellence (NICE) takes the view that at £5000-£15 000/QALY interventions are likely to be acceptable, at £25 000-£35 000/QALY special reasons would be needed to accept the intervention. Decisions are made on a case-by-case basis, and as the ICER increases the likelihood of rejection also increases [32].

Along with production efficiency, decision makers must consider affordability. A focus only on a threshold would lead to an infinite increase in costs. This is because many programmes, more than can be afforded, may fall below a threshold. The economic reality is that health resources are scarce and it may not be possible to increase the health care budget, if we recommend an option which increases costs, additional funds will have to be taken from elsewhere and redirected toward the new intervention. The cost of losing currently funded programs must be known before any final decision is made [33].

The results of economic evaluation are often not incorporated into decision making. Reasons for this include limited dissemination of the results, a lack of recognition of the implications of the results, poor understanding of the process of economic evaluation, a lack of belief in the results, and a lack of political power to enact change based on the results [14]. Where decisions have been made based on the results of economic evaluation, there are several factors taken into consideration. A cost-effectiveness threshold is applied, above which decisions are increasingly unlikely to be in favour of adopting the intervention and below which the intervention is usually adopted. Cost-effectiveness is considered in conjunction with the level of uncertainty surrounding the results and the burden of the disease under consideration. Social value judgements concerning equity, fairness, and what is considered to be good for society may also influence the decision [32].

It is clear that decisions in health care will always be made in the midst of a complex interplay of political, social and economic factors. However, economic evaluation is used to inform decisions, for example, the Australian breast and cervical screening programs were developed in conjunction with economic evaluation [34]. Whilst currently mostly restricted to pharmaceuticals, many jurisdictions now require economic evidence to accompany funding requests. With requirements for this evidence growing amidst scarce resources and fixed health budgets, it is reasonable to assume that such evidence will become an essential requirement for all interventions and therapies under consideration [35].

NICE in the United Kingdom is an organisation which uses economic evaluation and cost-effectiveness as the basis for decisions. The role of NICE is to provide recommendations concerning which new and existing technology should be funded by the National Health Service (NHS). These recommendations are legally binding adding significant weight to implementation [36]. The Pharmaceutical Benefit Advisory Committee (PBAC) performs a similar role in Australia for pharmaceuticals, and similarly the Medical Services Advisory Committee (MASC) looks at medical technologies and procedures. NICE has the advantage of being able to set its own agenda and prioritise evaluation, as well as successfully using a rapid response method, ideal when answers are required within short timeframes [37].

#### what else influences the decision to accept CAM?

Rigorous research and economic evaluation of CAM is a necessary step toward acceptance and adoption of these medicines and therapies by health care decision makers. Other issues, including historical and current public acceptance and demand, practitioner acceptance (particularly conventional practitioners), as well as political attitudes, will be influential in the decision to incorporate CAM into a comprehensive health care system. Conventional practitioners and decision makers have to date tended not to focus on CAM [38]. Different practitioner's beliefs and their exposure to and experience with CAM therapies all play a role, and in a sense, despite evidence of efficacy and effectiveness, CAM therapies have to repeatedly demonstrate their value [39]. Economic evaluation adds weight to the growing evidence base for CAM and will make it harder for CAM and its potential benefits to be overlooked in health care provision and practice decision-making.

## Conclusions

Economic evaluation is a decision-making tool for informing clinical practice and health policy (aiding informed decision-making regarding CAM and its integration into the health care system). The use of decision analytic modelling as a method of economic evaluation is a flexible method well suited to CAM as it does not rely solely on RCTs, but combines multiple data sources and can extrapolate beyond the limits of current clinical evidence. Efficacy and cost-effectiveness must be established and the results widely distributed in order for CAM to be extensively considered in healthcare decision-making. Information about economic evaluation is widely available, however, without any formal training CAM practitioners may be challenged in an attempt to utilise these economic evaluation methods. As with contemporary health research more generally, it is certainly important that multidisciplinary teams be deployed in order to share disciplinary insights and supplementary research knowledge. One example of how such a multidisciplinary approach is being facilitated at a national and international level is through initiatives of the Network of Researchers in the Public Health of Complementary and Alternative Medicine (NORPHCAM). NORPHCAM has established a health economics research stream helping to introduce and facilitate international collaborations between health economists, CAM practitioners and a range of other researchers interested in developing the economic evaluation of CAM. Whether examining the use of CAM alone or as an integrated component of contemporary health care provision, further consideration of economic evaluation as a research tool is required. This paper provides an impetus for those interested to pursue such a worthy goal.

## Competing interests

The authors declare they have no competing interests.

## Authors' contributions

EF completed first drafts. All authors contributed equally to all other aspects including conception, drafting and revising. All authors read and approved the final manuscript.

## Pre-publication history

The pre-publication history for this paper can be accessed here:

http://www.biomedcentral.com/1472-6882/10/66/prepub
